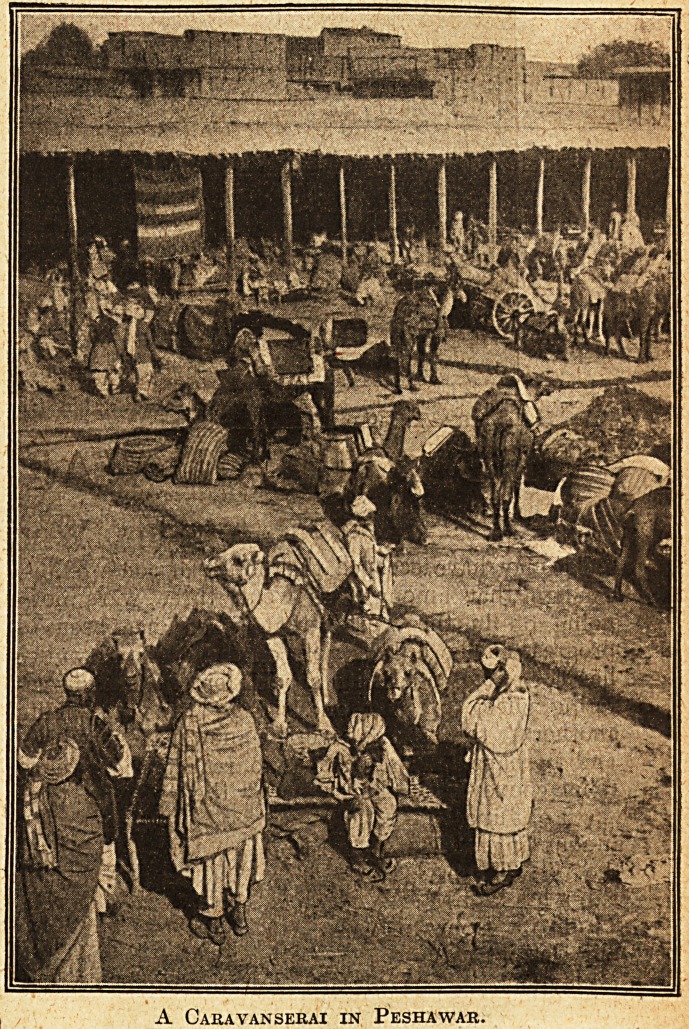# Medical Mission Work in Frontier Hospitals

**Published:** 1919-06-21

**Authors:** 


STAFF OF BRITISH GENERAL HOSPITAL, BANNU.

### THE AFGHAN WAR AND KHYBER PASS.

### Medical Mission Work in Frontier Hospitals.

IT may have escaped the notice of some of our readers that among the twenty-three wars now going on in the world, that which has been in progress in the hill districts on the Afghan frontier has been conducted with special ferocity and devastating effect. "Just one savage tribe attacking another" thinks the average newspaper reader, and perhaps fails altogether to realise the intense relief conveyed to thousands by the Reuter's telegram of June 16, announcing "In the Hazara, Peshawar, Bannu, Kohat and Derajat areas all is quiet."

Up in the outposts of civilisation on the Afghan frontier work is going on under the aegis of the Church Missionary Society which is too little known in this country. The source of its inspiration is the love of Christ. Its visible form of expression is found in the Medical Missions at Bannu and Peshawar.

The Mission Hospital at Bannu owed its initiation to Dr. Pennell, who some years ago put up a little building with mud-built walls as a temporary measure, little guessing that the fully-equipped hospital for which he longed would be built within a few years as a memorial to himself. The dangers and difficulties to which those who take part in these pioneer enterprises are exposed are illustrated by the Mahsud plot to kidnap Mrs. Pennell in 1915 and hold her as hostage until terms could be made with the Government. The plot was frustrated, and work in the memorial hospital where Mrs. Pennell took on the men's surgery increased till it included the care of over 1,600 in-patients and no fewer than 36,757 out-patient visits.

ONE MAN LEADING FIVE OTHERS TO THE HOSPITAL.

In 1917, owing to the unrest among the Mahsud tribes and their repeated outrages, an expeditionary force was despatched to quiet the district. Bannu became the base for the North Waziristan field force, and the Mission hospital was taken over by the military authorities. The Mission staff was employed on field-service duty and the equipment stores and drugs were found invaluable. For the first month the Mission staff carried on with little extra help, and then nursing sisters (Q.A.I.M.N.S.I.) and orderlies arrived. It now runs smoothly as a British general hospital with a hundred beds. The medical officers are the Mission doctors, including two women, and the Mission Indian workers serve with the dressers and orderlies.

The C.M.S. Hospital at Peshawar, founded in 1905, will always be connected with the name of Dr. Vernon Starr, whose murder on March 17, 1918, created so profound an impression. He was brutally stabbed early one morning by two men whom he believed to be patients and died within a few hours. A tireless worker and a devout servant of his Master, he was an ideal medical missionary, for he held both objects of the Mission in equal reverence. Assisted only by his wife herself a trained nurse, and by the nursing sister Miss Clarke, with a few partly-trained Indian helpers he had the medical and surgical work of the hospital entirely on his hands, together with its financial administra-

AN AFGHAN OUTSIDE SEAT.

tion, and brought it to a high state of efficiency. The building consists of blocks on three terraces, the lower being the out-patients' department, the middle terrace containing the wards for in-patients, and the residences of missionaries and staff lying on the highest. There are large waiting and consulting rooms for men and women, an out-patient operating theatre, and dressing-rooms. One of the in-patient blocks is on the lines of an Indian "serai," and is specially adapted for the trans-frontier patients who are accustomed to bring their relations with them in great force.

Mrs. Starr has given some vivid pictures of the cases received. As usual in the East, eye cases are very numerous, large numbers of patients being brought in during the winter months in camel caravans from far-distant regions for operation. One old Afridi woman brought her husband from afar with a broken leg, tied tight on a wooden bedstead fastened crossways to a camel's back. Cases of camel-bite are very common and present horrible septic wounds. "Lady-bites" are also to be encountered, one woman displaying a finger bitten clean off at the second joint "by another lady." The savage surgery from which the missionaries deliver their patients is illustrated by the man slightly bitten below the knee by a dog who was subjected by the native barber to a treatment which involved "ironing" with burning hot bricks from

A CARAVANSERAI IN PESHAWAR.

the knee to the thigh. He was admitted in a dying state to the hospital, but eventually recovered.

Doubtless, during the recent fighting the hospital at Peshawar, like that at Bannu, has transformed its character and taken on a military hue. On the restoration of peace, medical mission work will progress as it deserves to do with redoubled powers. Its great needs are workers. Will not some of the demobilised sisters now casting about for fresh fields remember the needs of the Church Missionary Society?

[NOTE.—Our readers cannot fail to be interested to learn that some members of THE HOSPITAL'S staff who have been in the fighting all through the War, went to India recently expecting escape from war conditions and some sport and relaxation, happily. On arrival they were sent right away to Peshawar, and there you are! Such is life in the British Empire in 1919. We wish our colleagues God-speed.—ED. THE HOSPITAL.]

## Figures and Tables

**Figure f1:**
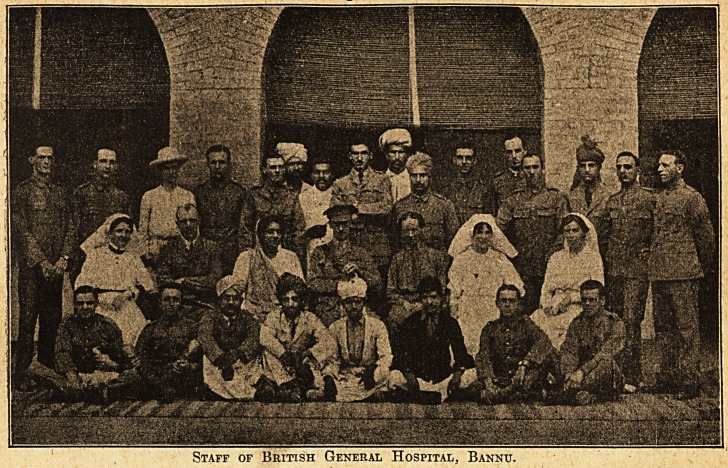


**Figure f2:**
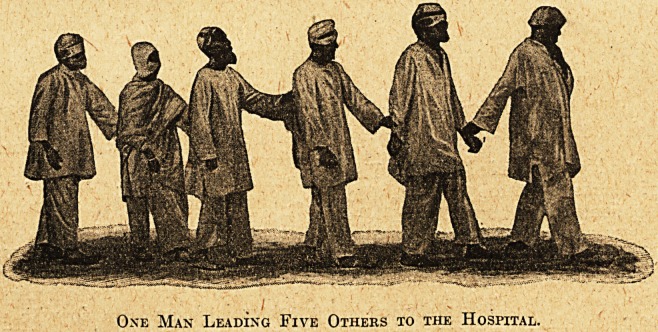


**Figure f3:**
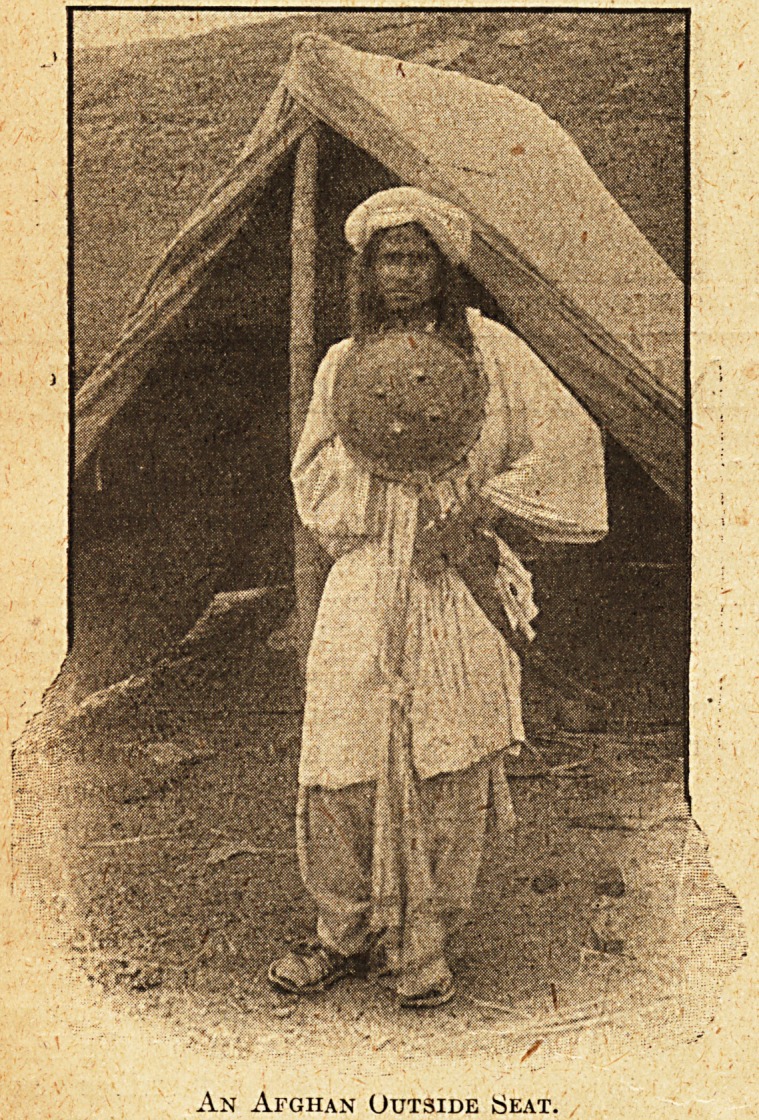


**Figure f4:**